# Crystal structure of Spa40, the specificity switch for the *Shigella flexneri* type III secretion system

**DOI:** 10.1111/j.1365-2958.2008.06293.x

**Published:** 2008-05-29

**Authors:** Janet E Deane, Stephen C Graham, Edward P Mitchell, David Flot, Steven Johnson, Susan M Lea

**Affiliations:** 1 Sir William Dunn School of Pathology, South Parks Rd, University of OxfordOX1 3RE, UK; 2 Division of Structural Biology, Wellcome Trust Centre for Human Genetics, Roosevelt Drive, University of OxfordOX3 7BN, UK; 3 European Synchrotron Radiation Facility6 Rue Jules Horowitz, 38043 Grenoble, France; 4 EPSAM, Keele UniversityStafforshire, ST5 5BG, UK; 5 European Molecular Biology Laboratory6 Rue Jules Horowitz, 38042 Grenoble, France

## Abstract

The pathogenic bacterium *Shigella flexneri* uses a type III secretion system to inject virulence factors from the bacterial cytosol directly into host cells. The machinery that identifies secretion substrates and controls the export of extracellular components and effector proteins consists of several inner-membrane and cytoplasmic proteins. One of the inner membrane components, Spa40, belongs to a family of proteins proposed to regulate the switching of substrate specificity of the export apparatus. We show that Spa40 is cleaved within the strictly conserved amino acid sequence NPTH and substitution of the proposed autocatalytic residue abolishes cleavage. Here we also report the crystal structure of the cytoplasmic complex Spa40_C_ and compare it with the recent structures of the homologues from *Escherichia coli* and *Salmonella typhimurium*. These structures reveal the tight association of the cleaved fragments and show that the conserved NPTH sequence lies on a loop which, when cleaved, swings away from the catalytic N257 residue, resulting in different surface features in this region. This structural rearrangement suggests a mechanism by which non-cleaving forms of these proteins interfere with correct substrate switching of the apparatus.

## Introduction

*Shigella flexneri* is the causative agent of bacillary dysentery in humans and is responsible for over a million deaths worldwide annually (Kotloff *et al*., 1999). Invasion of the colonic epithelium is dependent on a type III secretion system (T3SS) (Cossart and Sansonetti, 2004). T3SSs are found in many Gram-negative bacterial pathogens and serve as molecular injection devices to deliver bacterial virulence effector proteins into eukaryotic host target cells (Cornelis, 2006; Galan and Wolf-Watz, 2006; Blocker *et al*., 2008). The *Shigella* T3SS is encoded on a 31 kb fragment of a large virulence plasmid and consists of three major structural parts: a cytoplasmic region known as the ‘bulb’, a region spanning both bacterial membranes termed the basal body and an extracellular ‘needle’ (Blocker *et al*., 1999; Deane *et al*., 2006). The export apparatus comprises cytoplasmic and inner-membrane proteins that identify T3SS substrates and control the switching of substrate specificity during morphogenesis and upon host-cell contact (reviewed in Cornelis, 2006; Galan and Wolf-Watz, 2006). Following assembly of the basal body, early substrates are targeted to the apparatus, including the protein that polymerizes to form the hollow, extracellular needle. Upon completion of the needle, middle substrates (also known as translocators) are secreted that assemble at the tip of the needle and are involved in host-cell recognition and pore formation in the host-cell membrane. Following an activation signal, late substrates (also known as effectors) are targeted to the apparatus and translocated directly into host cells.

The *S. flexneri* protein Spa40 has been identified as a component of the basal body (Zenk *et al*., 2007) and is predicted to have a large, N-terminal transmembrane domain (Spa40_TM_) and a C-terminal cytoplasmic domain (Spa40_C_). It shares sequence homology and predicts membrane topology with YscU, an essential component of the secretion apparatus in *Yersinia* species (Allaoui *et al*., 1994). The cytoplasmic domain of YscU undergoes cleavage at a highly conserved NPTH sequence (Lavander *et al*., 2002) in a process that is suggested to be involved in the regulation of substrate specificity of the *Yersinia* T3SS (Edqvist *et al*., 2003). Substitution of the conserved Asn or Pro residues to Ala prevents cleavage of YscU (Lavander *et al*., 2002) and abolishes the export of translocators without affecting the export of effectors (Sorg *et al*., 2007). It is thought that cleavage of YscU results in a conformational change that allows the recognition of translocators, thus playing a role in determining the export hierarchy (Sorg *et al*., 2007).

T3SSs are genetically, morphologically and structurally related to the basal bodies of bacterial flagella (Blocker *et al*., 2003). The flagellar homologue of YscU, FlhB, plays a role in substrate specificity switching during flagella assembly (Hirano *et al*., 1994; Kutsukake *et al*., 1994; Williams *et al*., 1996; Fraser *et al*., 2003). The controlled switch between export states is believed to be mediated by conformational changes in the structure of the C-terminal cytoplasmic domain of FlhB. As is the case for YscU, this domain is consistently and specifically cleaved at the highly conserved NPTH sequence into two subdomains that remain tightly associated with each other (Minamino and Macnab, 2000a). The mechanism of FlhB cleavage has been analysed by Ferris *et al*. (2005) who provide evidence that the tertiary structure of FlhB plays a significant role in cleavage and propose that FlhB cleavage is an autocatalytic process.

Recently, the structures of the cleaved and non-cleavable forms of the cytoplasmic domains of the homologous proteins *Escherichia coli* EscU and *Salmonella typhimurium* SpaS were determined (Zarivach *et al*., 2008). These structures confirmed the autocatalytic mechanism proposed by Ferris *et al*. (2005) and provided the first structural insight into the subtle conformational change arising from the autocleavage event. Here we report the crystal structure of the *S. flexneri* Spa40_C_ complex and compare it with EscU_C_ and SpaS_C_. These structures highlight the tight association of the cleaved cytoplasmic subdomains and reveal that the conformational change upon cleavage involves only the movement of the PTH loop. The surface exposure of this loop and its orientation in the cleaved form suggest that it is the surface features in this region, rather than the cleavage event itself, that are important for binding partner proteins. Comparison of Spa40_C_ with the recent structures of EscU_C_ and SpaS_C_ as well as mapping of important functional mutations from several species provides insight into the potential roles of Spa40 in regulation of substrate secretion.

## Results and discussion

**Fig. 1:**
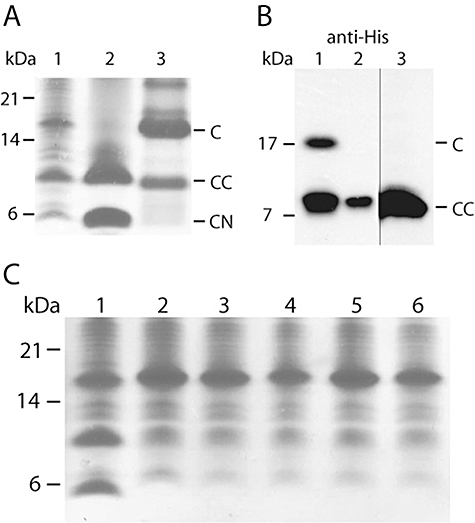
Cleavage and self-association of Spa40. A. Coomassie-stained SDS-PAGE of Spa40_C_ whole-cell lysate (lane 1), after native purification by affinity and size-exclusion chromatography (lane 2) and after purification under denaturing conditions (lane 3). B. Western blot using an antibody against the His_6_ tag of whole-cell lysate from overproduction of Spa40_C_ (lane 1) and Spa40_FL_ (lane 2). Western blot of the membrane fraction from overproduction of Spa40_FL_ (lane 3). C. Coomassie-stained SDS-PAGE of whole-cell lysate from overproduction of wild-type sequence Spa40_C_ (lane 1) and mutant forms of Spa40_C_: Asn→Ala (lane 2), Asn→Leu (lane 3), Asn→His (lane 4), Asn→Asp (lane 5), Asn→Gln (lane 6).

Whole-cell lysate from overproduction of the cytoplasmic domain of Spa40 (Spa40_C_) revealed three major bands (Fig. 1A, lane 1). Purification of the soluble fraction revealed that the two lower-molecular-weight bands co-elute upon purification, the third band being insoluble (Fig. 1A, lane 2). N-terminal sequencing of the purified complex revealed that the lower band corresponds to the N-terminal portion of Spa40_C_ starting at residue D207. The sequence for the upper band begins at P258 within the conserved NPTH sequence. Mass spectrometry data for the purified complex (6382 ± 2 and 10872 ± 2 Da) corresponded to the expected masses (6382 and 10871 Da) following cleavage of Spa40_C_ on the N-terminal side of Pro258 and confirmed that there was no modification to the newly formed termini, consistent with the cleavage mechanism proposed by Ferris *et al*. (2005) and Zarivach *et al*. (2008). These data confirm the identification of the lower and upper bands as Spa40_CN_ (residues 207–257) and Spa40_CC_ (residues 258–342) respectively, and establishes that Spa40_C_ is cleaved at the highly conserved NPTH sequence. As the C-terminal His-tag is only present on Spa40_CC_, co-elution from the Ni-NTA and size-exclusion columns established that Spa40_CN_ and Spa40_CC_ form a stable complex following cleavage. Uncleaved Spa40_C_ is not present in the soluble fraction but is found entirely in inclusion bodies. Purification of Spa40_C_ under denaturing conditions revealed that Spa40_CN_ did not co-elute with the Spa40_CC_ domain, identifying that the folded state of these proteins is required for their association (Fig. 1A, lane 3).

Western blot analysis of whole-cell *E. coli* lysate following overproduction of full-length Spa40 (Spa40_FL_) revealed a band corresponding to the same mass as the cleaved Spa40_CC_ domain identified in Spa40_C_ preparations (Fig. 1B, lanes 1 and 2). Following cell fractionation, this band was found to be enriched in the membrane fraction (Fig. 1B, lane 3), suggesting that the observed cleavage is maintained in the context of the full-length, membrane-localized protein. This result, using heterologous overproduction of Spa40_FL_ in *E. coli*, is in agreement with the observation that Spa40 isolated from preparations of the *S. flexneri* intact basal body has a molecular weight of approximately 33 kDa, representing the Spa40_TM_ plus Spa40_CN_ domains following cleavage of Spa40_CC_ (Zenk *et al*., 2007).

Cleavage at the conserved NPTH site is abolished by replacement of the Asn with Ala in the *Yersinia* and flagellar homologues of Spa40 (Lavander *et al*., 2002; Ferris *et al*., 2005). This substitution was generated in *Shigella* Spa40_C_ by site-directed mutagenesis and analysis of whole-cell *E. coli* lysate revealed that the lower bands representing the cleaved products are not present (Fig. 1C). Affinity purification, under native conditions, of Spa40_C(N257A)_ followed by N-terminal sequencing identified only the uncleaved Spa40_C_ (data not shown). Additional non-conservative substitutions with Leu or His and conservative substitutions to Asp or Gln also resulted in the production of only uncleaved Spa40_C_ (Fig. 1C). The absence of cleaved product for these mutants confirms the essential role of the Asn side-chain in the cleavage reaction.

Crystals were grown of the purified Spa40_C_ complex. Data sets were collected for two different *P*1 crystal forms, both containing one protein molecule in the asymmetric unit (Table 1). The structure of Spa40_C_ was solved by molecular replacement and refined against the higher-resolution data. The model comprises residues 237–257 of Spa40_CN_ (residues 207–236 were present in the construct but not visible in electron density) and 258–338 of Spa40_CC_ (the last four residues, 339–342, were not visible in electron density). The structure was refined using data to 2 Å with residuals *R*_work_ and *R*_free_ of 17.8% and 22.7% respectively (Table 1).

**Table 1:** Data collection and refinement statistics (values in parentheses are for the highest-resolution shell).

	Crystal form 1	Crystal form 2
Data collection		
X-ray source	ESRF ID23-2	ESRF ID23-1
Detector	MarMosaic 225 CCD	ADSC 315 CCD
Wavelength (Å)	0.873	1.040
Space group	*P*1	*P*1
Unit-cell dimensions		
a (Å)	24.6	25.0
b (Å)	30.5	30.8
c (Å)	32.0	32.1
α (°)	103.6	102.5
β (°)	110.1	111.0
γ (°)	104.8	94.3
Resolution limits (Å)	27–2.25 (2.37–2.25)	30–2.0 (2.11–2.00)
Measured reflections	6347	14093
Unique reflections	3481	5433
Completeness (%)	91.1 (85.1)	93.2 (93.3)
Multiplicity	1.8 (1.7)	2.6 (2.7)
*R*_merge_	0.098 (0.31)	0.109 (0.399)
*R*_pim_	0.098 (0.310)	0.084 (0.307)
Average I/σ(I)	5.4 (2.2)	3.8 (1.7)
Wilson *B*-value (Å^2^)	25.7	21.6
*Refinement*		
Resolution range (Å)		30–2.0 (2.05–2.00)
No. working set refl.s		5181 (414)
No. free set refl.s		252 (20)
*R*		0.178 (0.267)
*R*_free_		0.227 (0.292)
Number of atoms (protein/water)		806/29
Number of atoms with alternate conformations (protein/water)		3/0
r.m.s.d. bond length (Å)		0.010
r.m.s.d. bond angle (°)		1.105
Mean *B* factor (protein/water; Å^2^)		23.3/30.6
Ramachandran plot, residues in		
Favoured regions (%)		98.0
Allowed regions (%)		2.0

**Fig. 2:**
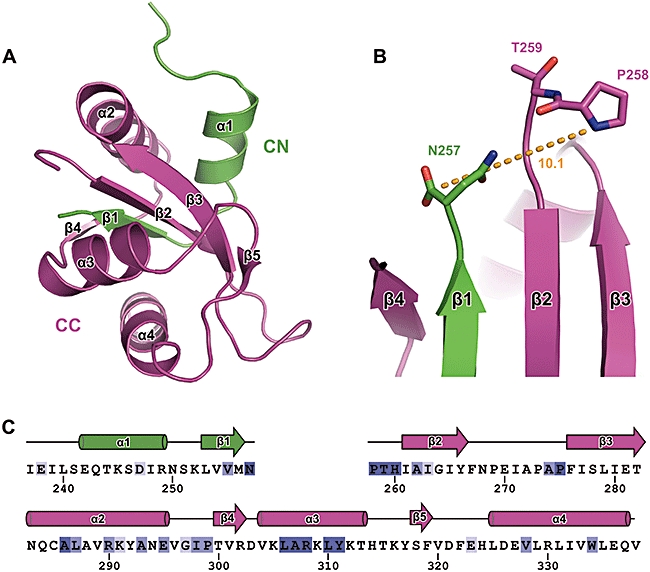
The structure of Spa40_C_. A. Ribbon diagram of Spa40_C_ showing Spa40_CN_ (green) and Spa40_CC_ (purple) with α-helices and β-strands labelled. B. Illustration of the flip of the cleaved PTH loop away from the catalytic N257. The distance between the carbonyl carbon atom of N257 and the amine nitrogen atom of P258 is shown in Å (orange). C. Sequence of Spa40_C_ coloured by conservation across species (dark blue being most conserved) using the multiple sequence alignment from Zarivach *et al*. (2008). The positions and labelling of α-helices (cylinders) and β-strands (arrows) are illustrated above the sequence.

The structure of Spa40_C_ consists of a central β-sheet surrounded by four α-helices (Fig. 2A). The structured region of Spa40_CN_ consists of a single α-helix (α1) followed by a single β-strand (β1) that lies at the centre of the structure and forms one strand of the five-stranded β-sheet. The structure of Spa40_CC_ starts at P258 and consists of four β-strands and three α-helices that wrap around β1 of Spa40_CN_. The NPTH sequence lies on a loop between β1 and β2, the PTH region having flipped away from N257, revealing that the cleavage of the N257-P258 peptide bond results in a conformational change to this loop region (Fig. 2B). P258 moves ∼10 Å from the catalytic asparagine residue and both P258 and T259 are less well-ordered than the surrounding residues, as indicated by higher isotropic temperature factors. The overall structure of Spa40_C_ and the movement of the PTH loop are very similar to that seen in the cleaved structures of the homologues from *E. coli*, EscU and *S. typhimurium*, SpaS (possessing ∼30% sequence identity, 1.26 ± 0.10 and 1.00 ± 0.09 Å r.m.s.d. over 92 Cα residues respectively) (Zarivach *et al*., 2008). The autocleavage mechanism requires a specific conformation of the NPTH loop, this conformation being stabilized by the correctly folded Spa40_C_ domain.

**Fig. 3:**
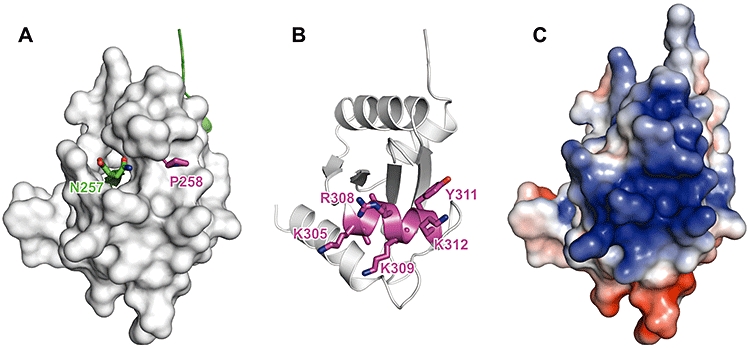
Surface features of Spa40_C_. A. The surface of Spa40_CC_ (white) and a ribbon representation of Spa40_CN_ (green) are shown. The side-chain atoms of N257 and P258 are shown as sticks. B. Ribbon diagram of Spa40_C_ in the same orientation as for A showing helix α3 (purple) and the side-chain atoms of residues K305–K312. C. Electrostatic surface of Spa40 showing the highly conserved positive patch (blue) surrounding the NPTH region. For calculation of electrostatics of those residues where the side-chain orientation could not be assigned because of a lack of electron density (R302, K305, F322, E323, Q337 and V338), the side-chains were modelled in the highest probability rotamer conformation that avoided clashes. Electrostatic surfaces were calculated using apbs (Baker *et al*., 2001) and are displayed on the molecular surface (plotted at ± 3 kT e^−1^).

The striking feature of these structures is that Spa40_CN_ forms part of the core of the fold with the conserved hydrophobic residues L253 to M256 (LVVM) completely buried by the surrounding Spa40_CC_ structure. For this reason, the cleavage at the NPTH site does not result in a large rearrangement of the domains but is restricted to a conformational change of the PTH loop only. Although cleavage does not result in a major rearrangement of the Spa40_CN_ and Spa40_CC_ domains with respect to each other, the structural changes in the NPTH loop region significantly alter this face of the molecule. Upon cleavage, the creation of charged terminal residues alone alters the properties of Spa40. However, the surface exposure of the NPTH loop and the movement of the cleaved PTH loop away from N257 result in several changes to the surface of Spa40_C_ (Fig. 3A). In particular, the side-chains of P258, T259 and H260 are exposed on the surface in an orientation that would not be possible in the uncleaved protein. The movement of the loop also exposes underlying residues that now form part of the new surface. Mutations that abolish cleavage will hold the PTH loop in a conformation that will bury these residues and thus alter the surface properties of Spa40_C_. Indeed, Zarivach *et al*. (2008) showed that the structure of the non-cleaving mutants of EscU can fold correctly and the only structural difference in these mutants is the orientation of the PTH loop. For these reasons, it is likely that it is not the act of cleaving the N–P bond but the result of that cleavage, which is the change in surface exposure of specific residues, that is critical. Therefore, the altered function of the non-cleaving forms of these proteins is probably the result of the loss of binding or recognition of other T3SS components.

The region from residue L306 to Y311 is highly conserved across all species and with the homologue from flagella, FlhB, possessing the consensus sequence LARXLY. This can be mapped to helix α3 which lies directly below the NPTH loop region (Fig. 3B). The hydrophobic residues in this region are buried and likely to be important for the correct positioning of the helix. In Spa40, the face of this helix that is exposed on the surface is composed of residues K305, R308, K309 and K312, resulting in a highly positively charged patch (Fig. 3C). The high conservation of residues involved in the positioning of this helix and the proximity of this charged patch to the NPTH loop suggest that it may play a role in the recruitment or binding of T3SS components. Although several of these charged residues are conserved in closely related species, such as *Salmonella*, only R308 is conserved in all Spa40 homologues and in FlhB. This may indicate that as species diverged, the core fold was conserved, but the surface features, and therefore the interaction faces, evolved to satisfy other requirements, such as specificity between T3SS and flagellar substrates in those species that possess both.

**Fig. 4:**
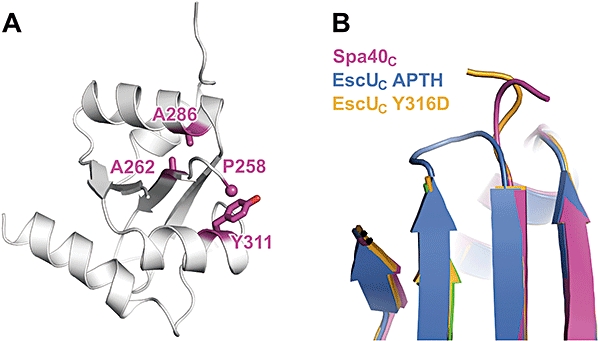
Mapping of mutations from *Yersinia* and flagellar homologues. A. Position of conserved residues that are altered by mutations that suppress a YscP/FliK knockout identified in *Yersinia* YscU and flagellar FlhB. B. Orientation of PTH loop in Spa40_C_ (purple) and the EscU mutant structures EscU_C__(APTH)_ (blue) and EscU_C__(Y316D)_ (orange).

In order to ensure correct assembly of the T3SS, the substrate specificity of the secretion apparatus must be switched upon completion of the needle, or hook in flagella. It is well established for both flagella and the *Yersinia* T3SS that this switch is regulated by two proteins. The first protein belongs to the Spa40 family (YscU in *Yersinia* and FlhB in flagella) and the second is responsible for determining the length of the needle/hook (Spa32 in *Shigella*, YscP in *Yersinia* and FliK in flagella). In the wild-type situation, interactions between the C-terminal domains of these proteins are thought to be responsible for the switching mechanism (reviewed in Cornelis, 2006; Ferris and Minamino, 2006). Functional knockouts of Spa32, YscP or FliK result in extended needle/polyhook structures (Hirano *et al*., 1994; Tamano *et al*., 2002; Journet *et al*., 2003). In both *Yersinia* and flagellar systems, mutations have been identified in the genes for YscU/FlhB that suppress the phenotype of the YscP/FliK knockout (Kutsukake *et al*., 1994; Williams *et al*., 1996; Edqvist *et al*., 2003). Several of these mutations affect residues that are well conserved across species and with flagellar FlhB. We have mapped the position of these mutations onto our structure of Spa40 (Fig. 4A). The YscU residues A268 and V292, equivalent to Spa40 A262 and A286, when mutated to Phe and Thr respectively, suppress the YscP knockout. It is clear from the structure that insertion of these larger side-chains would disrupt the packing of β2 and α2 in the region of the NPTH loop. It is likely that this perturbation would interfere with the folding of Spa40_C_ and so may affect the ability of the NPTH loop to adopt the conformation required for efficient cleavage. This is in agreement with the observation that the equivalent mutations in FlhB are resistant to the cleavage process (Minamino and Macnab, 2000a).

Another suppressor mutant identified in flagella and *Yersinia* is the mutation to aspartate of the highly conserved tyrosine residue within the LARXLY motif (Y317D in YscU, Y323D in FlhB, equivalent to Y311 in Spa40) (Williams *et al*., 1996; Edqvist *et al*., 2003). This residue lies on helix α3 and is partially exposed on the surface near the flipped P258 side-chain. This mutation does not inhibit cleavage and is likely to change the surface features of this region either directly or by interfering with the stability and/or orientation of the PTH loop. Indeed, Zarivach *et al*. (2008) showed that the equivalent change in EscU (Y316D) does alter the electrostatic surface features and also results in a change in the orientation of the PTH loop such that it points back towards the catalytic Asn (Fig. 4B). As this substitution does not inhibit cleavage at the NPTH site, this supports the hypothesis that it is the surface features in this region, rather than the cleavage event, that are important for function.

The mutations described above all interfere with the creation or characteristics of the NPTH surface region, but their effect must be independent of YscP/FliK as they can (partially) restore the function of a YscP/FliK knockout. There are several possible explanations for these observations. One is that the NPTH site may be the binding face for YscP/FliK and the alterations to this face mimic the bound form of YscU/FlhB, for example, by blocking the surface exposure of the NPTH region. Alternatively, the NPTH site may be involved, either directly or via an additional partner, in regulating the secretion of extracellular components of the apparatus. The cytoplasmic domains of the Spa40 homologues, YscU and FlhB, have been implicated in the recruitment of several early and middle substrates of the T3SS [YscI (Wood *et al*., 2008) and the translocators (Sorg *et al*., 2007) from *Yersinia*; FlgE and FlgD from flagella (Minamino and MacNab, 2000b)]. As needle assembly is proposed to involve the addition of needle monomers to the end of the growing needle and translocators are thought to assemble at the tip of this needle using a similar mechanism of binding (Deane *et al*., 2006; Johnson *et al*., 2007; Blocker *et al*., 2008), premature secretion of translocators would block the assembly of additional needle monomers. For this reason, an important step in correct needle assembly is the blockage of premature secretion of translocators. In this case, mutations to Spa40 that result in some recovery of needle length control, despite the absence of the length control protein, may act by interfering with this negative regulatory role for Spa40.

C-terminal truncations of FlhB have also been identified that partially suppress the phenotype of a FliK knockout. Zarivach *et al*. (2008) observed that the length of the C-terminal helix of EscU was similar to that of the FlhB truncation mutants while the SpaS C-terminal helix was longer. These observations, combined with the lack of an identified FliK homologue in *E. coli*, led Zarivach *et al*. (2008) to speculate that a shortened C-terminal helix may bypass the dependence of the needle length control mechanism on a FliK homologue. In our structure of Spa40, we observe a C-terminal helix of equivalent length to that of EscU. As the length of the *S. flexneri* T3SS needle is regulated by the FliK homologue, Spa32, and Spa40 possesses the shorter form of the C-terminal helix, the length of this helix is not crucial for regulation of T3SS needle length.

**Fig. 5:**
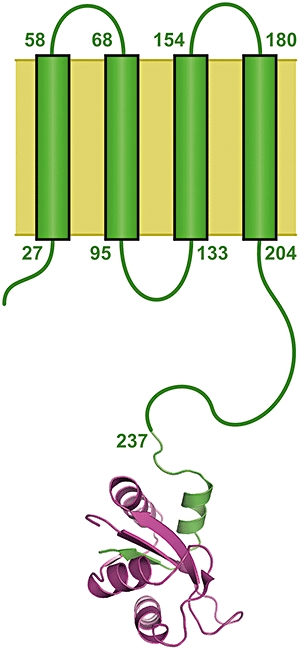
Relative size of the structured region of Spa40_C_ within the context of the full-length membrane protein. Positions of transmembrane helices are labelled as predicted by the Phobius web server (Kall *et al*., 2007).

The structures of EscU_C_, SpaS_C_ and Spa40_C_ all reveal that the N-terminal region of the cytoplasmic domain (CN), which links the folded domain to the inner membrane domain, is highly disordered (Fig. 5). Deletions or point mutations within this highly conserved region of EscU or FlhB abolish the secretion of both translocators and effectors (Fraser *et al*., 2003; Zarivach *et al*., 2008). Thus, mutations in this region affect the export capability of the apparatus rather than the substrate specificity. This observation suggests a distinct role for this flexible region in the secretion of substrates in general. The efficient secretion of substrates in both the pathogenic type III and flagellar systems is dependent on a soluble ATPase (FliI in flagella) (Woestyn *et al*., 1994; Fan and Macnab, 1996). In addition to their interactions with secretion substrates, the cytoplasmic domains of YscU and FlhB have been implicated in the recruitment of the ATPase regulatory complex [YscK–YscL–YscQ in *Yersinia* (Riordan and Schneewind, 2008), FliH and the general chaperone FliJ in flagella (Zhu *et al*., 2002)] and the ATPase itself [FliI in flagella, (Zhu *et al*., 2002)]. In this case, the changes within the CN domain may affect the correct positioning or movement of the Spa40_C_ domain and so alter its ability to correctly recruit other components to the secretion apparatus.

Several mutations have been identified within the first 30 residues of FlhB that can partially rescue the non-motile phenotype of a FliI (ATPase) knockout. This gain of function has been attributed to an increase in the probability of entry of flagellar proteins into the export gate (Minamino *et al*., 2003; Minamino and Namba, 2008). Thus, in addition to the proposed role of FlhB in recruitment of the ATPase complex to the secretion apparatus, these observations support a model whereby the FlhB family of proteins also plays a role in the blockage of secretion and identifies mutations that alleviate this block. It has been shown in both *Yersinia* and flagella that production of the cytoplasmic domain of YscU/FlhB exerts a dominant negative phenotype by blocking type III secretion (Fraser *et al*., 2003; Riordan and Schneewind, 2008). It remains uncertain whether the mechanism of this blockage involves sequestration of soluble components or localization of the cytoplasmic domain to the apparatus and direct blockage of the channel.

Following assembly of the extracellular components, the T3SS secretion apparatus must be negatively regulated in order to block secretion of late effectors prior to host-cell contact. This negative regulation is mediated, in part, by proteins belonging to the YopN family (Deng *et al*., 2005; Schubot *et al*., 2005). Functional knockouts of members of this family have no effect on needle formation, but result in enhanced effector secretion as well as altered secretion of translocators (Forsberg *et al*., 1991; Kubori and Galan, 2002; O'Connell *et al*., 2004). Interestingly, despite these proteins playing a role in the correct targeting of translocators and in the temporal regulation of effector secretion, the structures of the *Yersinia* and *Shigella* members of this family possess no structural homology with Spa40_C_ (Schubot *et al*., 2005; Deane *et al*., 2008). Thus, it is likely that the mechanisms of secretion regulation by the T3SS will differ at distinct stages of regulation.

In summary, we have shown that the *S. flexneri* homologue of the T3SS substrate switching protein, Spa40, is cleaved on the N-terminal side of P258 within the highly conserved NPTH sequence to produce a stable complex consisting of the subdomains, Spa40_CN_ and Spa40_CC_. The structure of this complex is similar to those of *E. coli* EscU and *S. typhimurium* SpaS, confirming that the CN region of these proteins forms part of the core of the fold. Cleavage at N257 creates new charged termini and results in the rearrangement of the surface-exposed PTH loop, altering the surface properties of Spa40_C_ in the vicinity of a conserved highly charged patch. The change in surface properties upon cleavage is likely to be critical for the correct binding of additional T3SS components. The mapping of several functional mutations identified in different regions of the *Yersinia* and flagella homologues onto our structure suggests possible mechanisms of action for this class of proteins in substrate switching and secretion regulation.

## Experimental procedures

### Protein production and purification

Constructs were made encoding the full-length protein (Spa40_FL_) and the cytoplasmic domain (residues 207–342, Spa40_C_). DNA fragments were produced by PCR (FLfwd 5′-AGGAGATATACCATGGCAAATAAAACAGAAAAGCCGACAC-3′, FLrev 5′-GTGATGGTGATGTTTATGAGTGTTTTCAACCTGCTCAAGC-3′, Cfwd 5′-TACCATGGATATGATGATGGATAAACAGGAG-3′ and Crev 5′-TGCTCGAGATGAGTGTTTTCAACCTGC-3′) and cloned into pOPINE (Berrow *et al*., 2007) for the Spa40_FL_ construct and pET28b (Novagen) for the Spa40_C_ construct to produce C-terminally His_6_-tagged proteins. Spa40_FL_ and Spa40_C_ were expressed in *E. coli* B834 (DE3) cells grown in LB media at 37°C until an A_600nm_ of ∼0.6 was reached, whereupon the cultures were cooled to 30°C and protein production was induced by the addition of 0.5 mM IPTG. After 4 h, cells were harvested by centrifugation (4000 *g*, 15 min, 4°C). The cell pellet was re-suspended in lysis buffer (20 mM Tris, pH 8.0, 500 mM NaCl and Complete EDTA-free protease inhibitor cocktail from Roche) and lysed using an Emulsiflex-C5 Homogeniser (Glen Creston, UK). Following centrifugation (20 000 *g*, 20 min, 4°C) the supernatant fraction was flowed over a 5 ml Ni-NTA Superflow column (Qiagen) at 1 ml min^−1^. Protein was eluted using a step gradient, with elution of Spa40_C_ at 300 mM imidazole. Fractions containing Spa40_C_ were further purified by size-exclusion chromatography using a HiLoad 26/60 Superdex 75 column (Amersham Biosciences) equilibrated with 20 mM Tris, pH 8.0 and 500 mM NaCl. Spa40_C_ was also purified under denaturing conditions by re-suspending the pellet following cell lysis in 20 mM Tris pH 8.0, 500 mM NaCl, 2 M Urea and 2% (v/v) Triton X-100. This suspension was centrifuged (15 000 *g*, 30 min, 4°C) and the pellet re-suspended in the same buffer without Triton X-100 and centrifuged again. This pellet was re-suspended in 20 mM Tris pH 8.0, 500 mM NaCl and 6 M GnCl and stirred overnight at 4°C. This suspension was centrifuged as before and the supernatant was applied to a 5 ml Ni-NTA Superflow column pre-equilibrated in 20 mM Tris pH 8.0, 500 mM NaCl and 8 M Urea and eluted using a step gradient as for the native purification except for the presence of 8 M Urea. Cells expressing Spa40_FL_ were grown as for Spa40_C_. To fractionate cells, the supernatant following cell lysis and removal of cell debris (20 000 *g*, 20 min, 4°C) was centrifuged to isolate the membrane fraction (94 000 *g*, 1.5 h, 10°C). Western blots were carried out using an HRP-conjugated antibody to the His_6_ tag (Qiagen).

### Crystallization

The purified complex of Spa40_CN_ with Spa40_CC_ was concentrated using Millipore Ultra-15 5k MWCO centrifugal filtration devices to 3.3 mg ml^−1^ and immediately put into crystallization trials. Initial crystallization conditions were obtained by sparse-matrix screening using the sitting drop vapour diffusion technique. Drops were prepared using an OryxNano crystallization robot (Douglas Instruments) by mixing 0.2 μl of protein (3.3 mg ml^−1^, 20 mM Tris pH 8.0, 500 mM NaCl) with 0.2 μl of reservoir solution and were equilibrated against 100 μl of reservoir solution at 20°C. Diffraction quality crystals in crystal form 1 grew in 3 weeks in condition P2-14 (0.1 M bisTris propane pH 6.5, 0.2 M NaBr and 20% w/v PEG 3350) of the PACT Premier screen (Molecular Dimensions). Crystals that diffracted to higher resolution in a different *P*1 crystal form were grown in condition P1-32 (0.1 M Hepes pH 7.0, 0.2 M NH_4_Cl and 20% PEG w/v 6000) of the PACT Premier screen. Crystals were cryoprotected in reservoir solution supplemented with 25% (v/v) glycerol for 15 s and flash-cryocooled by plunging into liquid nitrogen.

### Data collection

Initial diffraction data were collected on small plate-like crystals (50 μm × 20 μm × 5 μm) of form 1 of the Spa40_C_ complex at the microfocus beamline ID23-2 at the European Synchrotron Radiation Facility (ESRF), France (Table 1). Data were collected in three batches, each of 180° with 1.0° oscillations, from different regions of the same crystal using a helical-collection strategy, whereby the crystal is moved linearly between two points on the crystal as it is rotated, in order to minimize radiation damage effects (D. Flot *et al*., in preparation). This method was necessary because of the small size of the crystal and the number of images required for a complete data set in the space group *P*1. This data set was used for initial structure determination (below). Higher-resolution data were collected from optimized crystals in the second *P*1 crystal form at beamline ID23-1 (Nurizzo *et al*., 2006) at the ESRF and used for structure refinement. All data collection was performed at 100 K. Data were indexed and integrated in Mosflm (Leslie, 1992) and scaled with Scala (Evans, 1997) within the CCP4 program suite (CCP4, 1994).

### Structure determination

The structure of Spa40_C_ was solved by molecular replacement using the program phaser (McCoy *et al*., 2007) with a poly-serine search model generated using Chainsaw (CCP4, 1994; Schwarzenbacher *et al*., 2004) from the structure of the *E. coli* homologue EscU_C_ (PDB code 3BZL). Iterative cycles of refinement with REFMAC5 (Murshudov *et al*., 1997) and model building in coot (Emsley and Cowtan, 2004) were performed in consultation with the validation tools present in coot and with the MolProbity web server (Davis *et al*., 2007). Molecular figures were generated using PyMol (DeLano, 2002).

The atomic co-ordinates and structure factors have been deposited at the RCSB Protein Data Bank with Accession Code 2VT1.
